# New O- and N-N-Bridging Complexes of Tc(V), the Role of the Nitrogen Atom Position in Aromatic Rings: Reaction Mechanism, Spectroscopy, DTA, XRD and Hirshfeld Surface Analysis

**DOI:** 10.3390/ijms232214034

**Published:** 2022-11-14

**Authors:** Anton Petrovich Novikov, Mikhail Alexandrovich Volkov

**Affiliations:** Frumkin Institute of Physical Chemistry and Electrochemistry, Russian Academy of Sciences, Leninskii Prospect 31-4, 119071 Moscow, Russia

**Keywords:** technetium, pyridazine, triazole, dimethylpyrazole, pyrimidine, technetyl, XRD-analysis, Hirshfeld surface analysis, π-interactions, metal-nitrogen bond, supramolecular chemistry

## Abstract

In this work, O- and N-N-bridging complexes of technetium (V), previously known only for rhenium, were obtained for the first time. Tc(V) complexes with pyridazine (pyd), 1,2,4-triazole (trz), 3,5-dimethylpyrazole (dmpz) and pyrimidine (pyr) were obtained. In three complexes [{TcOCl_2_}_2_(μ-O)(μ-pyd)_2_], [{TcOCl_2_}_2_(μ-O)(μ-trz)_2_]·Htrz·Cl and [{TcO(dmpz)_4_}(μ-O)(TcOCl_4_)] two technetium atoms are linked by a Tc-O-Tc bond, and in the first two, Tc atoms are additionally linked by a Tc-N-N-Tc bond through the nitrogen atoms of the aromatic rings. We determined the role of nitrogen atom position in the aromatic ring and the presence of substituents on the formation of such complexes. For the first time, a reaction mechanism for the formation of such complexes was proposed. This article details the crystal structures of four new compounds. The work describes in detail the coordination of Tc atoms in the obtained structures and the regularities of the formation of crystal packings. The spectroscopic properties of the obtained compounds and their mother solutions were studied. The decomposition temperatures of the described complexes were determined. An assumption was made about the oligomerization of three-bridged complexes based on the results of mass spectrometry. Through the analysis of non-valent interactions in the structures, π-stacking, halogen-π and CH-π interactions were found. An analysis of the Hirshfeld surface for [{TcOCl_2_}_2_(μ-O)(μ-pyd)_2_], [{TcOCl2}2(μ-O)(μ-trz)_2_] and their rhenium analogues showed that the main contribution to the crystalline packing is made by interactions of the type Hal···H/H···Hal (45.4–48.9%), H···H (10.2–15.8%), and O···H/H···O (9.4–16.5%).

## 1. Introduction

Technetium is chemically and environmentally available as TcO_4_^–^ and exhibits pronounced oxidizing properties in reactions. Depending on the reducing conditions Tc(VII)O_4_^–^ may convert to stable/unstable final/intermediate species of Tc(V), Tc(VI) or Tc(III). In special cases, the products could be either of a cluster nature [1], or carbonyl derivatives [2,3] of Tc [2+, 2.5+ and 0] oxidation states. The close attention of researchers is attracted by Tc(V) and Tc(III) complexes; they play the most important roles in the preparative [4,5,6], radiopharmaceutical [7,8] and industrial reprocessing chemistry of Tc [9].

Great interest in technetyl (V) arose in connection with its use in nuclear medicine as a biologically active ^99m^Tc radiopharmaceutical based on the formation of Tc(V) coordination compounds [10,11,12,13,14,15]. Tc complexes with amine and imine ligands are the main models of Tc binding to amino acids, proteins, antibodies, etc. Tc(V) usually forms trans-[O_2_L_4_Tc]^+^ [10,16,17], [OL_n_Tc]^+^ [18,19], and [TcNL_n_] [20,21] where L is an N-containing organic ligand.

Many organic compounds based on heterocyclic molecules have bactericidal and cytotoxic characteristics, and they are frequently found in biological systems. Halogen atoms are frequently found in organometallic complexes, and the structure of the complexes might vary depending on the type of heterocyclic ligand used. There were no references to Tc(V) compounds with the same structure as the previously reported Re(V) compounds, in which the rhenium atoms were linked by three bridges, two of which formed a heterocyclic ligand [22,23,24,25,26]. The formation of Re(V) alkoxy compounds with heterocyclic ligands is also mentioned in the literature, though only a few examples have been described for technetium [27]. The association of these two types of compounds is discussed in works describing alkoxy-Re(V) compounds, as well as works describing the formation of Re(V) complexes with bridging ligands. Several groups have observed some general patterns in the formation of such molecules. The authors of Reference [28] propose that the production of Re-O-Re dimeric complexes occurs in the presence of water and that interaction with alcohols can result in alkoxy derivatives. Based on reactions involving Re(V) molecules, O=Re=O compounds are thought to constitute the final byproducts. Similarly, the authors of References [29,30] mention the production of dimeric and alkoxy derivatives of Re (V).

Understanding the patterns of interaction of technetyl (V) with heterocyclic bases of various structures can be used to predict the behavior of radiopharmaceuticals in living organisms, calculate quantum chemical interactions of technetium compounds, and plan further chemical studies. Weak intermolecular interactions are of particular interest from a theoretical point of view, the study of such interactions in pertechnetates and perrhenates of purines led to the discovery of a new type of chemical bond [31,32]. New methods of separating perrhenates and pertechnetates are based on non-valent interactions [33,34,35,36,37]. The study of the non-valent interactions contribution for various technetium compounds will allow for a deeper understanding of the impact of radiopharmaceuticals on living organisms [7] and the formation of non-trivial technetium complex compounds, similar to those described in References [4,5,16,38]. Information about weak interactions can also be useful for predicting the behavior of Tc-containing intermediates in catalytic and sorption processes on complex organic sorbents like those described in Reference [39].

This work is devoted to the synthesis and characterization of a new, previously known for rhenium, type of technetium compound. Four new coordination compounds were obtained in the work: **1**—[{TcOCl_2_}_2_(μ-O)(μ-pyd)_2_], **2**—[{TcOCl_2_}_2_(μ-O)(μ-trz)_2_]·Htrz·Cl, **3**—[{TcO(dmpz)_4_}(μ-O)(TcOCl_4_)], **4**—[TcO(pyr)_2_Cl_2_(OMe)], where pyridazine (pyd), 1,2,4-triazole (trz), 3,5-dimethylpyrazole (dmpz) and pyrimidine (pyr). For the compounds obtained, some data on thermal stability, electron absorption spec-troscopy of solutions, and infrared spectroscopy of the obtained powders are presented, as well as intermolecular interactions in crystals are considered by the Hirshfeld surface analysis. MALDY-ToF spectroscopy was performed on dried precipitates of mother liquor. All these methods, in combination with other experimental data presented in the paper, made it possible to develop a mechanism for the synthesis of Tc(V) organometallic complexes.

## 2. Results and Discussion

### 2.1. Structural Description of Tc(V) Complexes

The crystals of the compounds **1**, **3**, and **4** described below were mostly small-sized and poorly diffracted, and it seemed impossible to record the experiment with better quality even at low temperatures (100 K). 

**Figure 1:**
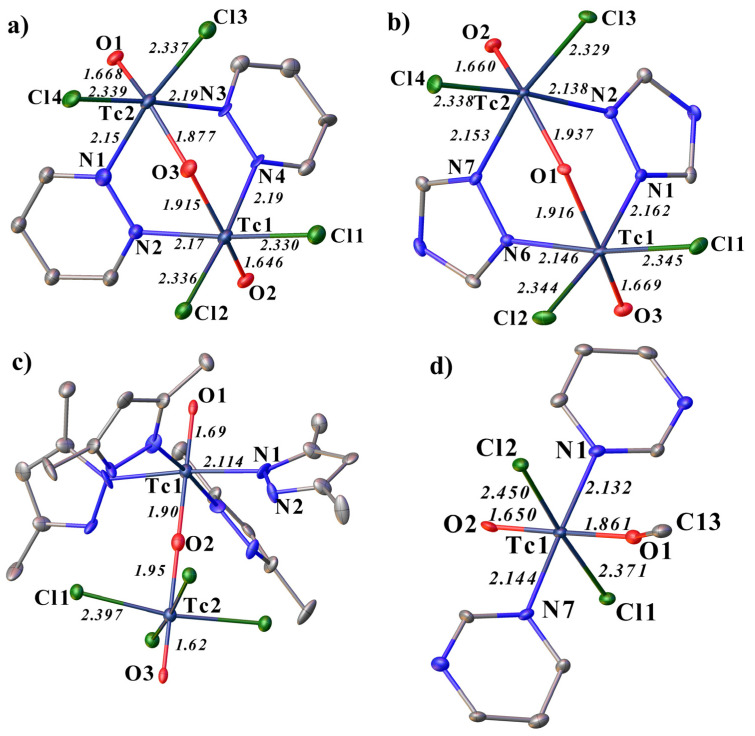
Molecular structure of **1** (**a**), **2** (**b**), **3** (**c**) and **4** (**d**) representing the coordination of the technetium atom with some bond lengths and labeling. Solvent molecules, counterions and H-atoms are omitted for clarity.

In all these compounds, the technetium atom exhibits an oxidation state of +5. In **1**, **2**, and **3**, the technetium atoms are linked to each other through an oxygen bridge. Moreover, in **1** and **2**, they are additionally linked by a Tc-N-N-Tc bond through the nitrogen atoms of the aromatic rings (Figure 1). In structure **2**, although the studied molecule of the complex is electrically neutral, the structure contains a triazolium cation and a chloride anion. All studied molecules of the complexes are electrically neutral. The Tc-N distances are close in **1** and **2** (in **1**, the Tc-N distances change from 2.15(2) to 2.19(2) Å, in **2** from 2.138(3) to 2.162(3) Å). The shortest Tc-N distance is observed in the complex with dimethyl pyrazole (2.11(2) Å). The N-Tc-N angles in **1** are 90°, and in **2** they are slightly different from 90° (86° and 87°). At **3** and **4**, the N1-Tc1-N1^1^ and N1-Tc1-N7 angles are 174° and 178°, respectively. In structures **1** and **2**, the Tc-O-Tc angles are equal to 128°, and in **3** this angle is 180°. The lengths of Tc-O bonds with bridging oxygen atoms are extended within one complex by 0.02–0.05 Å, the longest Tc-O bond is observed in **3** (1.95(7) Å), which may be related to another Tc2 environment. The lengths of Tc-O bonds with terminal oxygen atoms in **1**, **2**, and **4** are close (1.650(8)–1.67(2) Å). The largest elongation (1.69(4) Å) and shortening (1.62(4) Å) of the Tc-O bond is observed in **3**. The lengths of the Tc–Cl bonds in **1** and **2** are close, in **3** this bond is slightly longer (from 2.332(9)–2.345(1) Å to 2.397(5) Å). It should be noted that, in **4**, the length of the Tc1-Cl1 bond is close to **1**–**3** (3.371(3) Å), and the Tc1-Cl2 bond is slightly lengthened (2.450(3) Å).

The N-N bond length in **1**–**3** varies from 1.33(3) to 1.40(3) Å; in **4** the N···N distance is 2.38(2) Å. An increase in the distance between the nitrogen atoms and a change in the angle between them can explain the formation of a different type of complex, which is different from **1** and **2**. In **3**, a different type of complex is formed, apparently due to the presence of a proton at one of the nitrogen atoms, as in a similar rhenium analogue [40].

The angles and distances in **1** are close to those described previously for the rhenium analog with pyridazine [24,25]. At the same time, for tetrazole, **2** compounds were not similar, as has been described in the literature, neither with a rhenium atom, nor with a technetium atom. Additionally, the compounds with dimethylpyrazole described for rhenium have a different geometry and structure [22,23,41,42,43].

**Figure 2:**
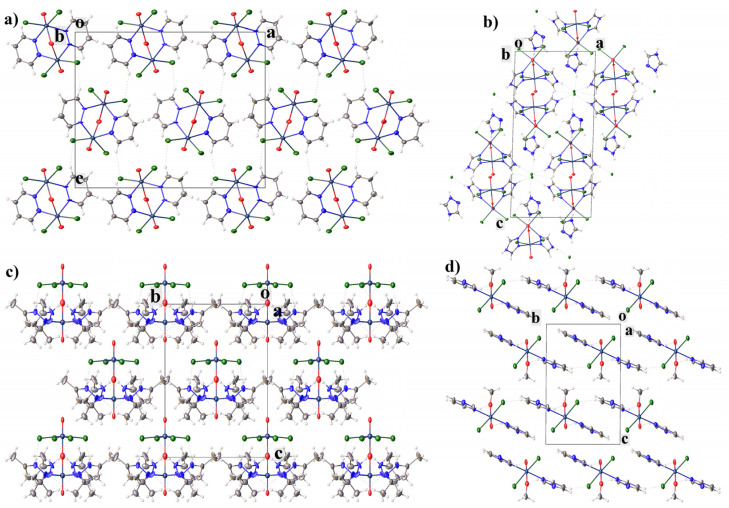
Crystal packing of **1** (**a**), **2** (**b**), **3** (**c**) and **4** (**d**) showing the complexes’ layers.

**Figure 3:**
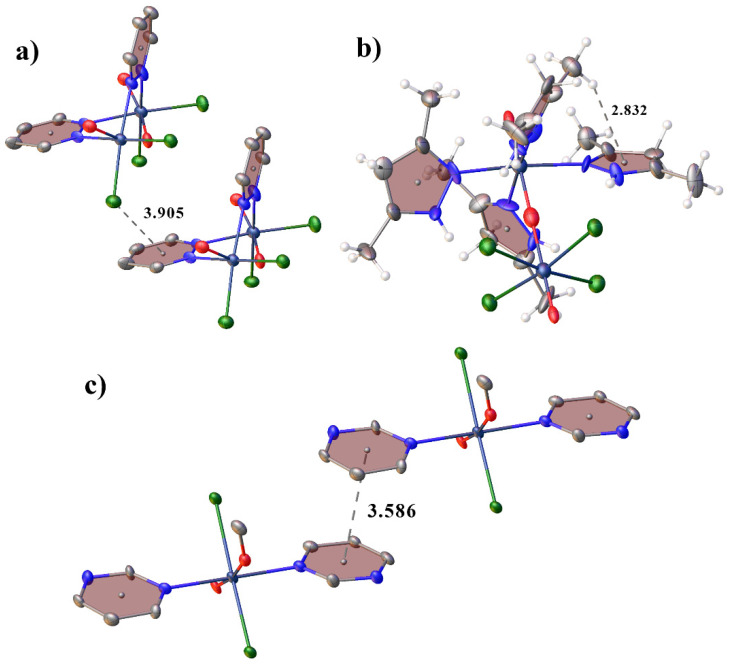
View showing halogen-π in **1** (**a**), CH-π in **2** (**b**) and π-stacking interactions in **3** (**c**). H-atoms are omitted for clarity in (**a**,**b**).

In **1**, only weak hydrogen bonds of the C-H···Cl type are present between the molecules of the complex. In **2**, due to the presence in the structure, in addition to the molecule of the complex, charged cation and anion molecules, a more complex system of hydrogen bonds is formed than in **1**. The molecules of the complex are interconnected through chloride anions by H-bonds of the N-H···Cl type and are connected to triazolium cations by hydrogen bonds of the type C-H···Cl (Figure 2). In **3**, unlike **1** and **2**, intramolecular H-bonds of the N-H···Cl and C-H···N type are formed. In **4**, intermolecular hydrogen bonds of the C-H···O type are formed. In **4**, the molecules of the complex are additionally interconnected by a π-stacking interaction between C2N1C6C5C4N3 and C8N7C12C11C10N9^1^ (symmetry code: *x*, 1 + *y*, *z*) rings (angle: 5.6(4)°, centroid-centroid distance: 3.586(7) Å, shift distance 0.84(1) Å), which is absent in **1**–**3** [16,44,45] (Figure 3a). However, in **3**, the methyl hydrogen of dimethylpyrazole participate in intramolecular CH-π interaction (the distances between the hydrogen atoms and the centers of the aromatic rings are shorter than 3 Å (2.83(1) Å)) [16,46,47,48,49]. Additionally, in **1** there are halogen-π interaction with Cl···ring-center distance 3.90(1) Å and Cl···C distance 3.35(3) Å (which is shorter than the sum of van der Waals radii equal to 3.45 Å) [50,51,52,53,54,55]. Crystal packing in all compounds can be represented as layered.

### 2.2. Hirshfeld Surface Analysis

Hirshfeld surface (HS) analysis is based on the division of the electron density in a crystal. The Hirshfeld surface covers the molecule and determines the volume of space in which the electron density of the promolecule exceeds the density of all neighboring molecules [56]. This method can be used to analyze different types of non-valent interactions [32,57,58,59,60,61].

**Figure 4:**
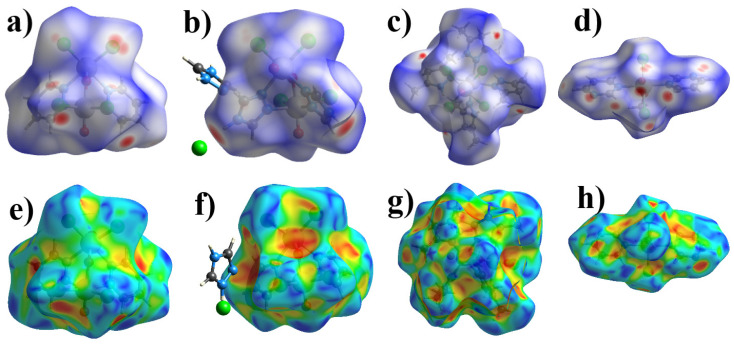
HS mapper over *d*_norm_ for **1** (**a**), **2** (**b**), **3** (**c**) and **4** (**d**) and HS mapper shape-index of **1** (**e**), **2** (**f**), **3** (**g**), and **4** (**h**) to visualize of intermolecular interactions in crystals.

The Crystal Explorer 21 [62] program was used to analyze non-valent interactions in crystals using the HS analysis. The donor-acceptor groups are visualized using a standard (high) surface resolution and *d*_norm_ surfaces (Figure 4a–d). Red spots on the *d*_norm_ surface correspond to contacts shorter than the sum of van der Waals radii, such as hydrogen bonds, π-stacking or CH-π interactions and blue ones, respectively, are longer than the sum of van der Waals radii. For the additional analysis of π-stacking interactions, shape-index surfaces were constructed for all the complexes. Only **4** has characteristic red and blue triangles on the shape-index surface corresponding to the π-stacking interaction, which are absent for **1**–**3** (Figure 4e,f).

**Figure 5:**
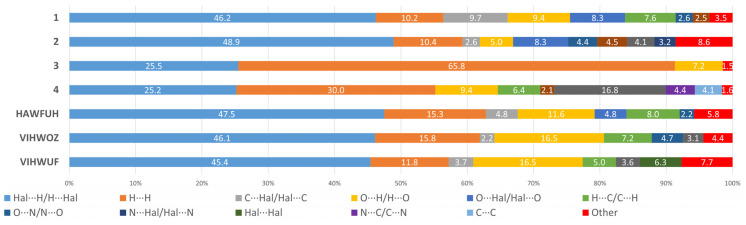
Percentage contributions of contacts to the Hirshfeld surface in structures **1**–**4** and analogues taken from CSD. HAWFUH: (µ2-oxo)-bis(dibromo-bis(pyridazine)-oxo-rhenium) acetone solvate [24]; VIHWOZ: (µ2-Oxo)-bis(µ2-pyridazine)-tetrachloro-dioxo-di-rhenium(v) benzene solvate [25]; VIHWUF: (µ2-Oxo)-bis(µ2-pyridazine)-tetrabromo-dioxo-di-rhenium(v) acetonitrile solvate [25].

We have analyzed the HS for analogues of the **1** and **2** compounds. Since no compounds like **1** and **2** are known for technetium (as well as for manganese), we decided to compare the contribution of non-valent interactions in **1**–**4** with rhenium analogs of **1**–**2** found in the Cambridge Structural Database [63]. With rhenium atoms, as mentioned above, only analogues with pyridazine, which connects rhenium atoms with an N-N bond, were found [24,25]. In all structures found, apart from complex molecules, solvent molecules such as acetone (HAWFUH: (µ2-oxo)-bis(dibromo-bis(pyridazine)-oxo-rhenium) acetone solvate [24]), benzyl (VIHWOZ: (µ2-Oxo)-bis(µ2-pyridazine)-tetrachloro-dioxo-di-rhenium(v) benzene solvate [25]), or acetonitrile (VIHWUF: (µ2-Oxo)-bis(µ2-pyridazine)-tetrabromo-dioxo-di-rhenium(v) acetonitrile solvate [25]) crystallized. However, at the same time, for **1**–**2** and for analogues, the main contribution to intermolecular interactions is made by contacts of the Hal···H/H···Hal type (Figure 5). The largest contribution of this type of interaction is observed for **2** (48.9%). In **3** and **4**, the share of this type of interactions is almost the same (25.5 and 25.2%) and almost two times less than in **1** and **2**. Van der Waals interactions of the H···H type make almost the same contribution to **1** (10.2%) and **2** (10.4%), but much more to the rhenium analogs. The greatest contribution to intermolecular interactions in **3** is made, in contrast to **1** and **2**, by H···H interactions (65.8%). At the same time, the proportion of this type of interaction in 4 is also significant (30.0%) and more than in **1** and **2**. For **1**, in contrast to other complexes, contacts of the C···Hal/Hal···C type (which are usually responsible for π-halogen interactions) make a large contribution, which may be due to the absence of solvent molecules or other ions in the structure. The contacts of the O···H/H···O type play an important role in non-valent interactions in rhenium analogs (16.5% and 11.6%), and account for only 5% in **2** and 9.4% in **1**. Contacts of the O···N/N···O type make a significant contribution to **4** (16.8%), in contrast to the other compounds. In **4**, contacts of the C···C type appear, which are responsible for the pi-stacking interaction. The contacts of the O···Hal/Hal···O type make the same contribution to **1** and **2** (8.3%), in the HAWFUH only 4.8%, while the rest are practically absent. It is worth mentioning that Br···Br-type contacts appear in VIHWUF, which are absent in **1** and **2** (these contacts are usually responsible for halogen bonds). Contacts that contribute less than 2% are not considered in Figure 5.

### 2.3. Spectroscopic Studies

**Figure 6:**
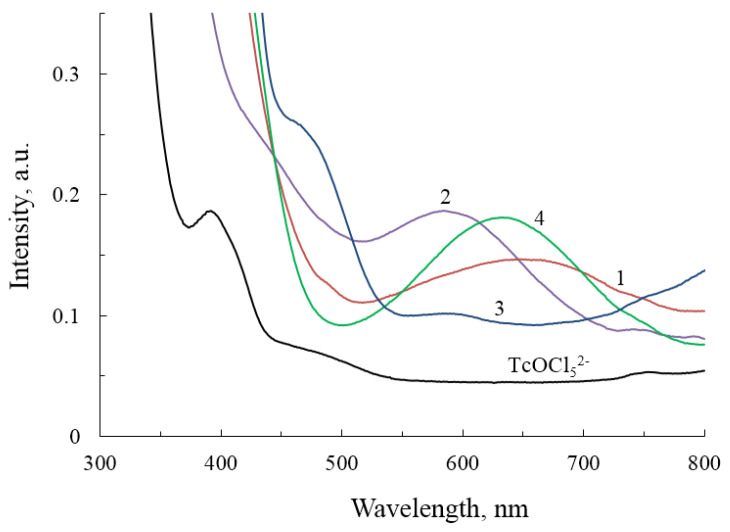
Electron absorption spectroscopy of mother methanol solutions of complexes: Compound **1**−curve 1; compound **2**−curve 2; compound **3**−curve 3; compound **4**−curve 4; and stock solution (NH_4_)_2_TcOCl_5_. The concentration of technetium in the solutions was equal and amounted to approximately 0.01 M.

Electron absorption spectroscopy of a methyl solution of NH_4_TcO_4_ in concentrated hydrochloric acid (Figure 6, curve 1) showed the presence of bands at 482, 414, 396, and 296 nm, indicative of Tc compounds in the reduced Tc(V) form, with an error of ±7 nm. An additional band at 245 ± 2 nm is found in the spectrum, which is characteristic of Tc(VII). UV-Vis spectroscopy of acetone and tetrahydrofuran solutions containing Tc(V) compounds was performed to clarify the peculiarities of the behavior of technetium compounds in the context of the reaction of complex compound synthesis with the participation of alcohols. Unlike methanol, all spectral bands were implicit; in the visible spectrum region, intense absorption occurs in the range from 500 to 350 nm, which is typical for Tc(V). Figure 6 illustrates the curves taken in a methanol/acetone solution. The addition of equimolar amounts of heterocyclic ligands to acetone solutions of technetium compounds (using 2,5-dimethylpyrazole and 1,2,4-triazole as examples) did not result in a band shift, indicating the absence of a complex formation reaction in the first minutes after the ligand was added.

During the initial minutes of the reaction, broad absorption bands in the range of 587–650 nm were detected in the dilute stock solutions of compounds **1**, **2**, **3**, and **4** by UV-Vis spectroscopy. Bands of the original compound Tc(V) (Figure 6) cannot be detected; the available bands corresponding to technetium halide complexes are displaced to the low-energy region and range from 400 to 500 nm. For complex **1**, two bands at 491 and 652 nm are recorded (Figure 6, curve 1); for complex **2**, two bands at 445 and 595 nm (curve 2); for complex **3**, two bands at 470 and 600 nm (curve 3); for complex **4**, one band was recorded with a peak at 635 nm (curve 4). The literature describes UV-Vis spectroscopy for compounds similar to complexes **1** and **2** obtained for Re(V). The spectra [22] likewise exhibits a broad band at 645 nm. The authors of [22] attribute the band around 645 nm to the significant character of the ligand field (5d_xy_ → d_xz_, d_yz_) [64,65]. Spectroscopy of compound **3** displays a wave similar to TcO_2_Cl_4_ halide complexes, as well as a band in the longer wavelength region similar to TcO_2_L_4_ compounds, where L is an N-containing ligand; a comparable spectrum was recorded for TcO_2_(Im)_4_^+^ compounds [16].

Based on the data presented, it can be concluded that technetium compounds X_n_Tc^V^_2_(μ-O)(μ-N-N)_2_ have absorption in the 600-650 nm range. However, if the molecule is not doubled by bridging ligands, the absorption wave shifts to a shorter wavelength region. It is impossible to interpret the results, since the heterocyclic compounds used in the work absorb intensely in the near UV range. The design of curves 1 and 2 suggests that distinct structural products from those isolated in the form of crystals may develop.

The extinction coefficients for compounds **1**, **2**, **4** were calculated from absorption bands in the range 600 ± 50 nm. For compound **3**, were calculated from the absorption band at 470 nm. Technetium concentrations were equal in all cases (*c* = 0.01 M), only one cell (*l* = 10.001mm) participated in the experiment. The calculated extinction coefficients for compounds **1**, **2**, **3**, and **4** (M^−1^ cm^−1^) were: 3.47, 9.89, 15.63, and 10.38, respectively.

Infrared spectroscopy of the obtained complexes showed the presence of multiple bands corresponding to organic heterocycles. The IR spectra of the complexes are shown in Supplementary Materials Figure S2. The intense absorption near 1100 cm^−1^, a feature of stretching vibrations of the C-N bond and stretching vibrations of C-H bonds in the range of 2800 to 3050 cm^−1^ are also present in the spectra of all compounds. The C-C bond of aromatic heterocycles exhibits strong absorption bands in the range of 1570–1625 cm^−1^ in the spectra of compounds **1**, **3**, and **4**. Stretching vibrations of the N-N bond may be responsible for the low-intensity absorption bands in the complexes **1** and **2** spectra between 1400 and 1500 cm^−1^. Compounds **3** and **4**’s spectra exhibit absorption bands at 1430 and 1470 cm^−1^, respectively, which indicate the CH_3_ group’s bending vibrations. Compound **4** has an absorption line of stretching vibrations of the C-O bond at 1020 cm^−1^ in its spectra. N-H vibrations are indicated by a broad peak in the 3300–3450 cm^−1^ region, which is only observed in the spectra of compound **2**. Additionally, all spectra can exhibit the long-wavelength waves of lingering water. Table S16 in the Supplementary Materials contains a complete list of peaks.

The Tc-N and Tc-Cl bond vibrations could not be detected on the spectra since the region specific to these vibrations (200–500 cm^−1^) was uninformative. All of the reported compounds’ spectra have distinct bands for Tc=O vibrations in the 790–950 cm^−1^ region [18,66].

### 2.4. MALDI-ToF Analysis of the Mother Liquor of ***1***

**Figure 7:**
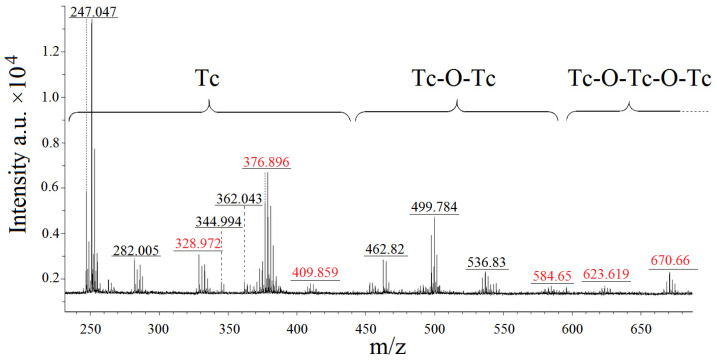
MALDI spectroscopy of the stock solution of compound **1**.

**Figure 8:**
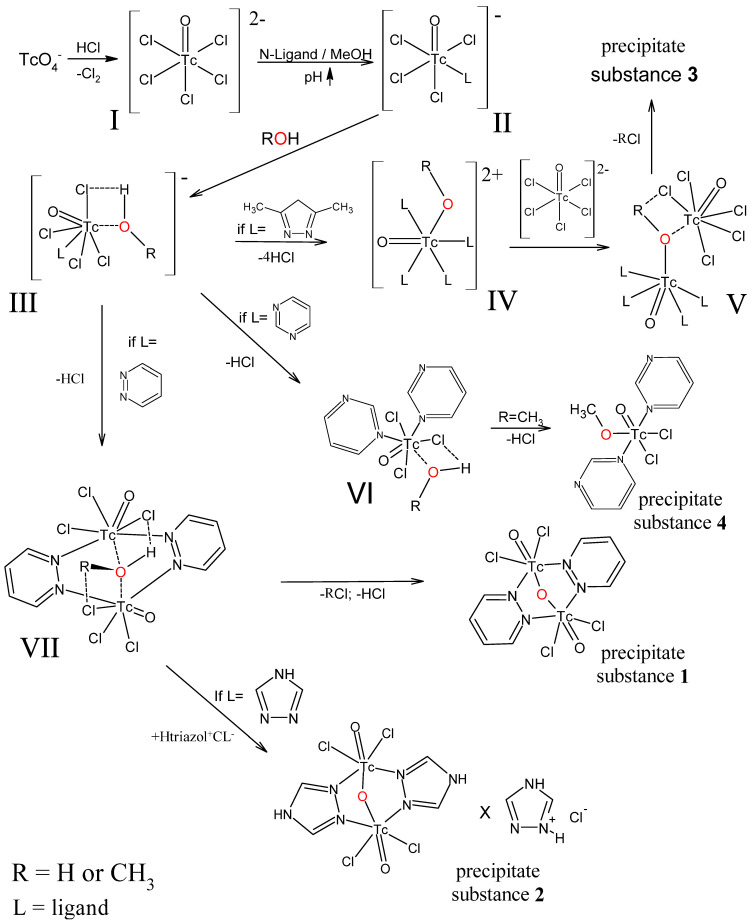
Proposed mechanism for the formation of compounds **1**, **2**, **3** and **4**.

Mass spectrometry was performed on the dried residues of component 1 stock solutions. The mass spectrum contains peaks with a maximum mass of 970 a.m.u., which, based on isotope distribution, correspond to the pyridazine resinification side processes and are not depicted in Figure 7. Peaks corresponding to chlorine and oxo derivatives of organic pyridazine fragments generated during laser desorption of the material under study are recorded with a high degree of probability in the mass range up to 251 a.m.u., with the exception of peaks in the region of 247 a.m.u. Peaks with proposed suitable formulas and structures are labelled as black in Figure 8 and are reported in the Supplementary Materials (Table S17). Red-labelled peaks were not interpreted and were excluded from the table.

According to the quantity of technetium atoms, the peaks in the spectrum were split into three groups. Complexes with only one technetium atom in their structure belong to the first category, which spans the 247–440 a.m.u. range. Peaks 247.047 a.m.u. (calc. 246.05) and 282.005 a.m.u. (calc. 281.98) correspond to particles with the gross formulas TcO_2_ClN_2_C_4_H_5_ and TcO_2_Cl_2_N_2_C_4_H_5_, respectively. Since each of these particles contains one heterocyclic fragment, it is possible that the formation of particles with only one heterocyclic fragment in their structure is one of the initial steps in the reaction for compound **1**. The gross formulas for the peaks at 344.94 a.m.u. (calc.344.97) and 362.043 a.m.u. (calc.361.99) are TcOCl_2_N_4_C_8_H_8_ and TcO_2_Cl_2_N_4_C_8_H_9_, respectively. The particles include two heterocycles, and the structures differ by one OH fragment.

The second group has a mass range of 450–590 a.m.u., and the associated particles have two technetium atoms joined by ligand and/or oxygen bridges. The fragments with peaks 462.82 (calc. 462.75), 499.784 (calc. 499.777), and 536.83 (calc. 536.79) likely preserve the Tc=O fragment and include one heterocycle each. With this assumption, we may assign the gross formulas Tc_2_O_5_Cl_3_N_2_C_4_H_4_, Tc_2_O_5_Cl_4_N_2_C_4_H_4_ and Tc_2_O_5_Cl_5_N_2_C_4_H_6_.

The third cluster of peaks is between 600 and 700 a.m.u. in mass, and the particles in this cluster include three technetium atoms connected by distinct types of bridges. The interpretation of heavy peaks is problematic; nevertheless, based on the nature of the isotopic distribution, fragments with a mass more than 600 a.m.u. can be presumed to have three technetium atoms in their structure. Three-bridge complexes Tc(V) can apparently oligomerize through ligand bridges.

### 2.5. Proposed Mechanism for the Formation of Complexes

The major anion generated during the TcO_4_^−^ reduction reaction in concentrated hydrochloric acid is the TcOCl_5_^2−^ specie I, which is typical of the transition metals in the +5-oxidation state (Figure 8). The addition of a heterocyclic base solution changes the pH of the medium and initiates hydrolytic activities with the creation of specie II. A methanol or water molecule is oriented towards specie II through a chlorine atom via a Cl···H hydrogen bond, while the ligand’s distortion of specie II can create conditions for nonvalent interactions of the oxygen atom with the metal atom and the formation of specie III. It should be noted that an excess of water can cause complete hydrolysis of technetium-containing specie and the formation of colloidal suspensions. The structure of the ligand influences the subsequent reaction with a heterocyclic base. Interaction with 2,5-dimethylpyrazole results in the substitution of three chlorine atoms and the production of Tc-N bonds, as well as the elimination of hydrochloric acid and the formation of the Tc-O link. Hydrolytic activities involving 2,5-dimethylpyrazole that result in the synthesis of specie IV appear to proceed swiftly; this can explain the presence of Tc(V) compounds in solution in diverse chemical forms, resulting in the formation of specie V. The formation of an oxygen bridge apparently occurs with the elimination of MeCl or HCl, this stage of the process is the rate-limiting one. Experiments have shown that the interaction of technetyl halide complexes with five-membered heterocycles can give several products of various structures, which are not discussed in this paper.

In the case of six-membered heterocycles, if the heteroatoms in the cycle are not conjugated, the formation of ligand bridges does not occur. Compound **4** is insoluble in alcohols. Hydrolytic reactions stop when only two chlorine atoms are replaced by pyrimidine. Since there is no delayed methyl chloride production stage, precipitation occurs relatively quickly. It is reasonable to expect that using a six-membered pyrazine heterocycle as a ligand will result in the creation of a compound similar to **4**. In the presence of conjugated heteroatoms in the cycle, such as pyridazine, Tc-N-N-Tc bridges and specie VII form. In the case of the formation of a complex, paired molecule, the formation of bridging oxygen and the elimination of HCl or MeCl become possible, which leads to a fairly rapid precipitation.

For 1,2,4-triazole, the process is seen in various ways. However, due to compound **2**’s low solubility, the equilibrium shifts towards the reaction product. However, the crystallization process takes longer than expected, and is likely caused by extra non-valent interactions of the nitrogen atom in position **4**, and as a result, the substance crystallizes alongside 1,2,4-triazole hydrochloride.

The formation of the described compounds leads to the conclusion that the mechanism of TcO_2_(Im)_4_^+^ imidazole compound formation proposed in Reference [16] can only be correct at the stages of reduction of Tc(VII) with thiourea. Based on the suggested mechanism for compound **3**, it can be assumed that the solvent also plays a role in the formation of TcO_2_(Im)_4_^+^ compounds.

### 2.6. DTA-Analysis

**Figure 9:**
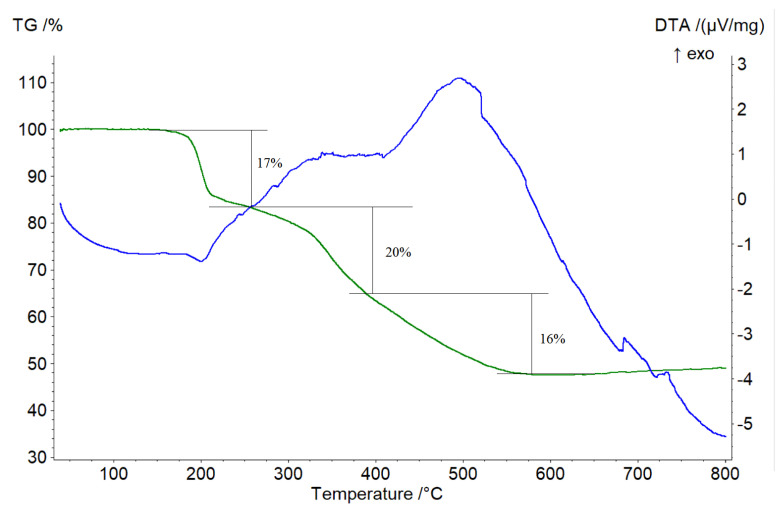
TG−DTA decomposition data of TcO(Pyr)_2_Cl_2_OMe in the Ar−H_2_ atmosphere.

The active phases of complexes **1**, **2**, and **3** decomposition occur in the range of 250–525 °C, according to TG-DTA analysis data. According to pXRD data, the thermolysis of all the compounds described in the study in an argon-hydrogen mixture atmosphere resulted in metallic technetium and an unidentified phase that contains technetium, oxygen, chlorine, and carbon atoms, according to X-ray fluorescence spectroscopy data. The decomposition temperatures of compounds **1**, **2** and **3** are 283 °C, 225 °C and 185 °C, respectively (Figure 9). Compounds **1**, **2**, and **3** lost 35%, 51%, and 55% of their total weight. Unfortunately, despite the relatively substantial weights of complexes **1–3** samples collected for thermal analysis (5 mg each); the data obtained were uninformative. Thermal analysis data for compounds **1** and **2** are provided in Figures S3 and S4 in the Supplementary Materials.

The active phase of compound **4** decomposition occurs in the temperature range of 180–530 °C, according to TG-DTA analysis data. The extrapolated temperature of the decomposition starts at 180 °C. Depending on the shape of the TG curve, at least three partially overlapping stages of the decomposition process can be distinguished. A weight loss of around 17% is seen in the 180–215 °C temperature range, and the decomposition process proceeds with heat absorption with a maximum in 200 °C. Further heating results in a slow decrease in mass to a temperature of 317 °C, which could be attributed to residual effects from the initial stage of decomposition, but no energy effects appear on the DTA curve. The second stage of decomposition occurs in the range of 320–380 °C, the weight loss is approximately 20%, while an unexpressed exothermic effect is observed on the DTA curve. The third phase of decomposition proceeds in the temperature range of 380–530 °C and is accompanied by heat emission. Further heating resulted in sample mass stabilization. During heating, the total weight loss was 53%. According to pXRD analysis, the residue was primarily composed of metallic technetium.

## 3. Materials and Methods

*Caution!* ^99^Tc is a β-emitter (*A* = 635 Bq/μg [67], *E_max_* = 290 keV); appropriate shielding and manipulation technics were employed during the synthesis and in all manipulations.

Pirimydine, 2,5-dimethilpyrazole, pyridazine, triazole were purchased from Sigma-Aldrich (Darmstadt, Germany). All reagents and solvents were qualified chemically pure and were not subjected to further purification.

The ammonium pertechnetate (ISOTOP RF) used in the work was recrystallized from bidistilled water (R ≥ 18 MΩ). The resulting white crystalline powder was especially pure and spectrally pure.

### 3.1. Synthesis of Complexes

Finely milled dry NH_4_TcO_4_ was dissolved in a minimum amount of concentrated hydrochloric acid (12 M). The resulting solution was evaporated to half at room temperature in the vacuum of an oil pump, while chlorine was intensively released from the solution. The residual greenish-yellow solution was diluted with methanol in a ratio of 2:1, and the appropriate heterocyclic ligand methanol solutions were slowly added to the resulting mixture under vigorous stirring until a saturated green color was formed. During the synthesis of compounds **1** and **4**, a precipitate began to form 2–3 min after the addition of the first drops of the ligand solution. For compound **2**, the formation of a precipitate began 12 h after the start of the reaction. Compound **4** was formed only upon evaporation of the mother liquor to half its volume. The resulting compounds were washed with methanol and air dried. The reaction yields were calculated relative to Tc as follows: **1**, **4**—95%, **2**—75%, and **3**—62%.

The elemental analysis calculated/found (%): For **1** Tc—36.11/37.73, C—17.54/17.81, N—10.23/10.05; for **2** Tc—31.24/32.65, C—11.38/11.43, N—19.91/19.85; for **3** Tc—25.55/26.67, C—31.03/31.1, N—14.47/14.2; for **4** Tc—26.23/27.26, C—28.67/28.9, and N—14.86/14.74.

The initial technetium and ligand solutions were diluted 20 times with methanol to create crystals **1** and **4** acceptable for scXRD analysis. After mixing, a barely perceptible green tint developed, and the crystals started to form 50–70 min later.

### 3.2. Single-Crystal XRD Analysis

The crystal structure of all synthesized substances was determined by X-ray structural analysis using an automatic four-circle area-detector diffractometer Bruker KAPPA APEX II with MoKα radiation. The cell parameters were refined over the entire data set, together with data reduction using SAINT-Plus software [68]. Absorption corrections were introduced using the SADABS program [69]. The structures were solved using the SHELXT-2018/2 program [70] and refined by full-matrix least squares on F^2^ in the anisotropic approximation for all non-hydrogen atoms (SHELXL-2018/3 [71]). Atoms H, bounded to CH- and NH- groups, were placed in geometrically calculated positions with isotropic temperature factors equal to 1.2 *U_eq_*(C, N) and 1.5 *U_eq_*(C) for CH_3_-groups. The H atoms in NH- groups in **2** were objectively located from the difference Fourier synthesis and refined with isotropic temperature factors equal 1.2 *U_eq_*(N). Structure **1** was refined as an inversion twin. Tables and figures for the structures were generated using Olex2 [72].

Crystal data, data collection, and structure refinement details are summarized in Table 1. All other crystallographic parameters of the structures are indicated in Tables S1−S15. The atomic coordinates were deposited at the Cambridge Crystallographic Data Centre [63], CCDC №° 2213501-2213504 for **1**–**4**. The Supplementary crystallographic data can be obtained free of charge from the Cambridge Crystallographic Data Centre via www.ccdc.cam.ac.uk/data_request/cif (accessed on 10 October 2022).

### 3.3. Spectroscopic Analysis

The IR spectrum of the compounds was registered at Nicolet IR200 FT-IR from the 2 mg sample pressed as a finely grounded mixture with 100 mg KBr and pressed at 50 kg/cm^2^.

UV-visible spectroscopy was studied using the Cary 100 Scan in the range from 900 to 300 nm. A 10 mm quartz cuvette was used for measurements. The cuvette width was 10.001 mm.

### 3.4. MALDI-ToF—Analysis

For the study, we used a mass-spectrometer Ultraflex II (Bruker) in the reflective mode on positive ions without the use of a matrix with an accelerating voltage of 25 keV. Desorption was performed by an Nd:YAG laser beam (λ = 355 nm). The interpretation of the obtained spectra and the identification of individual peaks were carried out using the FlexAnalysis-3.3 program.

### 3.5. Thermal Analysis

We performed thermal gravimetry with simultaneous differential thermal analysis (TG-DTA) using a Netzsch STA Jupiter 449 F3 thermoanalytical complex. Heating was carried out at a rate of 10 °C/min in the temperature range 40–1000 °C. Al_2_O_3_ crucibles and W-Re sample carrier were used; the atmosphere was the Ar-1.5% H_2_ mixture with a purity of ω(Ar + H_2_) = 99.999%. 

### 3.6. Elemental Analysis

The chemical composition of the compounds was determined on a sample of 50 mg each. The technetium in the compounds was determined by liquid scintillation on a Tri-Carb 3180 TR/SL instrument (PerkinElmer, Rodgau, Germany), using a HiSafe 3 scintillator; the measurement error did not exceed 5%. C, N, O were determined using the EA 3000 EuroVector analyzer (EuroVector, Pavia PV, Italy), the measurement error was not more than 10%. The contents of chlorine and hydrogen in the compounds was not measured.

## 4. Conclusions

Four new complex compounds of Tc(V) with heterocyclic compounds of various structures were synthesized and characterized: [{TcOCl_2_}_2_(μ-O)(μ-pyd)_2_], [{TcOCl_2_}_2_(μ-O)(μ-trz)_2_]·Htrz·Cl, [{TcO(dmpz)_4_}(μ-O)(TcCl_4_O)] and [Tc(pyr)_2_Cl_2_O(OMe)]. In the electronic absorption spectra of binuclear complexes Tc(V) showed that the presence of ligand bridges shifts absorption waves to longer wavelengths compared to mononuclear complexes. The absorption waves of Tc(V) coordination compounds with heterocyclic ligands are in the range of 550–650 nm. Based on the results of mass spectroscopy, it can be assumed that the interaction of Tc(V) halide complexes with heterocyclic compounds, having conjugated nitrogen atoms in their structure, can lead to the formation of oligomers Tc(V) complexes with the masses of complexes over 600 a.m.u. The decomposition temperatures for the obtained compounds **1**, **2**, **3** and **4** were established: 283 °C, 225 °C, 185 °C and 180 °C, respectively.

The interaction of Tc(V) compounds with heterocyclic N-containing ligands usually leads to the formation of [O=Tc=O]^+^ compounds; however, compounds of various structures can be formed depending on the position of the heteroatoms. Heterocycles having two or more conjugated nitrogen atoms in their structure lead to the formation of additional ligand bridges that prevent the formation of O=Tc=O compounds. Six-membered heterocycles having non-conjugated heteroatoms in their structure in an alcoholic solution lead to the formation of Tc(V) alkoxy compounds with two coordinated heterocyclic ligands. Apparently, the formation of Tc(V) complexes with heterocyclic ligands requires the presence of water or alcohols in the solution. The synthesis of substituted complexes proceeds faster with five-membered heterocycles, which leads to the formation of complexes with technetium atoms surrounded by ligands of various natures.

In the crystal structures obtained in this work, it was shown that the Tc-N distances vary from 2.11 to 2.19 Å and do not depend on the mode of coordination of the heterocycle to the Tc atom. The largest Tc-N distance is observed for the complex with pyd. The molecules of the complexes are bound together into crystal structures, as a rule, due to weak hydrogen bonds(C-H···Cl/O) and other non-valent interactions (π-stacking, halogen-π and CH-π). However, if there are cations and anions in the structure, binding occurs due to stronger H-bonds(N-H···Cl) through cations and anions. In [{TcO(dmpz)_4_}(μ-O)(TcOCl_4_)] intramolecular H-bonds of the N-H···Cl and C-H···N type are formed. The analysis of non-valent interactions by the Hirshfeld surface method for [{TcOCl_2_}_2_(μ-O)(μ-pyd)_2_], [{TcOCl_2_}_2_(μ-O)(μ-trz)_2_] and their comparison with rhenium analogs showed that, regardless of the halogen, metal atom, or the presence of other molecules in the structures, contacts the Hal···H/H···Hal (45.4–48.9%) type.

## Figures and Tables

**Table 1:** Crystal data and structure refinement for structures **1**–**4**.

Identification Code	1	2	3	4
Empirical formula	C_8_H_8_Cl_4_N_4_O_3_Tc_2_	C_6_H_10_Cl_5_N_9_O_3_Tc_2_	C_20_H_36_Cl_4_N_8_O_3_Tc_2_	C_9_H_11_Cl_2_N_4_O_2_Tc
Formula weight	545.98	631.30	774.37	376.12
Temperature/K	100(2)			
Crystal system	orthorhombic	monoclinic	tetragonal	triclinic
Space group	*Pna*2_1_	*P*2_1_/*n*	*I*4	*P*-1
a/Å	17.929(2)	10.4766(9)	10.059(6)	6.168(3)
b/Å	6.1567(9)	8.4060(7)	10.059(6)	8.617(4)
c/Å	14.726(2)	21.6695(18)	15.055(13)	13.054(6)
α/°	90	90	90	89.553(17)
β/°	90	91.572(3)	90	89.505(19)
γ/°	90	90	90	69.338(16)
Volume/Å^3^	1625.5(4)	1907.6(3)	1523(2)	649.2(5)
Z	4	4	2	2
ρ_calc_g/cm^3^	2.231	2.198	1.688	1.924
μ/mm^−1^	2.368	2.176	1.294	1.519
F(000)	1048.0	1216.0	780.0	372.0
Crystal size/mm^3^	0.3 × 0.12 × 0.05	0.22 × 0.09 × 0.06	0.06 × 0.05 × 0.04	0.39 × 0.12 × 0.04
Radiation	MoKα (λ = 0.71073)			
2Θ range for data collection/°	8.494 to 54.986	8.326 to 59.998	9.078 to 59.994	9.37 to 59.982
Index ranges	−23 ≤ h ≤ 23, −7 ≤ k ≤ 7, −19 ≤ l ≤ 18	−14 ≤ h ≤ 14, −11 ≤ k ≤ 9, −30 ≤ l ≤ 30	−14 ≤ h ≤ 13, −13 ≤ k ≤ 13, −21 ≤ l ≤ 20	−7 ≤ h ≤ 8, −12 ≤ k ≤ 11, −18 ≤ l ≤ 18
Reflections collected	18191	32465	3234	10884
Independent reflections	3664 [R_int_ = 0.1657, R_sigma_ = 0.1277]	5544 [R_int_ = 0.0925, R_sigma_ = 0.0779]	1764 [R_int_ = 0.1930, R_sigma_ = 0.3190]	3691 [R_int_ = 0.1534, R_sigma_ = 0.2125]
Data/restraints/parameters	3664/1/162	5544/0/239	1764/1/83	3691/0/165
Goodness-of-fit on *F*^2^	1.025	1.023	0.941	0.986
Final R indexes [I >= 2σ (I)]	R_1_ = 0.0691, wR_2_ = 0.1416	R_1_ = 0.0426, wR_2_ = 0.0734	R_1_ = 0.0971, wR_2_ = 0.1829	R_1_ = 0.1046, wR_2_ = 0.2348
Final R indexes [all data]	R_1_ = 0.1262, wR_2_ = 0.1680	R_1_ = 0.0659, wR_2_ = 0.0805	R_1_ = 0.2371, wR_2_ = 0.2429	R_1_ = 0.1907, wR_2_ = 0.2894
Largest diff. peak/hole/e Å^−3^	2.42/−1.45	0.97/−0.88	1.39/−1.28	3.04/−2.23
Flack parameter	0.5(2)		0.06(19)	

## Data Availability

The data presented in this study are available on request from the corresponding author.

## Supplementary Materials

The following supporting information can be downloaded at: https://www.mdpi.com/article/10.3390/ijms232214034/s1.
