# Supplementary material for: New O- and N-N-Bridging Complexes of Tc(V), the Role of the Nitrogen Atom Position in Aromatic Rings: Reaction Mechanism, Spectroscopy, DTA, XRD and Hirshfeld Surface Analysis
Source: Int J Mol Sci. 2022 Nov 14;23(22):14034. doi: 10.3390/ijms232214034 (PMC9696901; doi:10.3390/ijms232214034)
Supplement: Supplementary file 1 [file ijms-23-14034-s001.zip › ijms-2006176-supplementary.pdf]

Supporting Information

New O- and N-N-bridging complexes of Tc(V), the role of the position of the nitrogen atom in aromatic rings: reaction mechanism, spectroscopy, XRD and Hirshfeld surface analysis

Anton Petrovich Novikov*, Mikhail Alexandrovich Volkov

Frumkin Institute of Physical Chemistry and Electrochemistry, Russian Academy of Sciences, Leninskii prospect 31-4, 119071 Moscow, Russia
* Correspondence: tony.novickoff@yandex.ru

Table S1. Bond Lengths for **1.**

Atom	Atom	Length/Å		Atom	Atom	Length/Å
Tc1	Cl2	2.336(6)		N3	N4	1.33(3)
Tc1	Cl1	2.330(7)		N3	C8	1.32(3)
Tc1	O2	1.646(18)		N4	C5	1.32(3)
Tc1	N4	2.19(2)		C6	C7	1.41(3)
Tc1	O3	1.915(17)		C6	C5	1.45(3)
Tc1	N2	2.17(2)		C8	C7	1.37(3)
Tc2	Cl3	2.337(6)		C2	C3	1.36(3)
Tc2	Cl4	2.339(6)		C2	C1	1.37(3)
Tc2	N3	2.19(2)		N1	N2	1.40(3)
Tc2	O1	1.668(17)		N1	C4	1.35(3)
Tc2	O3	1.877(17)		C3	C4	1.38(3)
Tc2	N1	2.15(2)		N2	C1	1.31(3)

Table S2. Bond Angles for **1.**

Atom	Atom	Atom	Angle/°		Atom	Atom	Atom	Angle/°
Cl1	Tc1	Cl2	91.7(2)		O3	Tc2	N1	77.9(8)
O2	Tc1	Cl2	100.1(7)		N1	Tc2	Cl3	171.4(7)
O2	Tc1	Cl1	100.3(7)		N1	Tc2	Cl4	90.3(6)
O2	Tc1	N4	85.0(8)		N1	Tc2	N3	90.0(8)
O2	Tc1	O3	157.5(7)		N4	N3	Tc2	118.3(17)
O2	Tc1	N2	86.3(8)		C8	N3	Tc2	120.8(17)
N4	Tc1	Cl2	174.1(5)		C8	N3	N4	121(2)
N4	Tc1	Cl1	90.4(6)		N3	N4	Tc1	118.3(16)
O3	Tc1	Cl2	95.3(5)		C5	N4	Tc1	120.6(16)
O3	Tc1	Cl1	95.5(5)		C5	N4	N3	121(2)

O3	Tc1	N4	79.1(7)		Tc2	O3	Tc1	128.1(7)
O3	Tc1	N2	78.0(8)		C7	C6	C5	112(2)
N2	Tc1	Cl2	87.4(6)		N3	C8	C7	121(2)
N2	Tc1	Cl1	173.4(6)		C8	C7	C6	122(2)
N2	Tc1	N4	89.9(8)		C3	C2	C1	118(2)
Cl3	Tc2	Cl4	91.9(2)		N2	N1	Tc2	118.1(15)
N3	Tc2	Cl3	86.9(6)		C4	N1	Tc2	124.7(18)
N3	Tc2	Cl4	174.2(6)		C4	N1	N2	117(2)
O1	Tc2	Cl3	101.8(7)		N4	C5	C6	123(2)
O1	Tc2	Cl4	99.8(7)		C2	C3	C4	118(2)
O1	Tc2	N3	86.0(9)		N1	N2	Tc1	117.3(15)
O1	Tc2	O3	158.8(8)		C1	N2	Tc1	123.6(18)
O1	Tc2	N1	86.0(9)		C1	N2	N1	119(2)
O3	Tc2	Cl3	93.6(5)		N1	C4	C3	124(2)
O3	Tc2	Cl4	94.1(5)		N2	C1	C2	124(2)
O3	Tc2	N3	80.3(7)					

Table S3. Torsion Angles for **1.**

A	B	C	D	Angle/°		A	B	C	D	Angle/°
Tc1	N4	C5	C6	175.2(16)		N4	N3	C8	C7	-1(3)
Tc1	N2	C1	C2	175.1(18)		C8	N3	N4	Tc1	-175.3(16)
Tc2	N3	N4	Tc1	6(2)		C8	N3	N4	C5	2(3)
Tc2	N3	N4	C5	-175.9(17)		C7	C6	C5	N4	1(3)
Tc2	N3	C8	C7	176.9(16)		C2	C3	C4	N1	-5(4)
Tc2	N1	N2	Tc1	6(2)		N1	Tc2	O3	Tc1	50.3(12)
Tc2	N1	N2	C1	-176.8(17)		N1	N2	C1	C2	-2(4)
Tc2	N1	C4	C3	-179.7(17)		C5	C6	C7	C8	0(3)
Cl3	Tc2	O3	Tc1	-128.1(11)		C3	C2	C1	N2	0(4)
Cl4	Tc2	O3	Tc1	139.7(11)		N2	N1	C4	C3	4(4)
N3	Tc2	O3	Tc1	-41.8(12)		C4	N1	N2	Tc1	-177.1(17)
N3	N4	C5	C6	-2(3)		C4	N1	N2	C1	0(3)
N3	C8	C7	C6	0(3)		C1	C2	C3	C4	3(3)
O1	Tc2	O3	Tc1	9(3)						

Table S4. Bond Lengths for **2.**

Atom	Atom	Length/Å		Atom	Atom	Length/Å
Tc1	Cl1	2.3447(10)		N12	N11	1.365(5)
Tc1	Cl2	2.3435(11)		N12	C13	1.297(6)

Tc1	O1	1.916(3)		N9	C8	1.337(5)
Tc1	O3	1.669(3)		N9	C10	1.343(5)
Tc1	N1	2.162(3)		N14	C15	1.344(6)
Tc1	N6	2.146(3)		N14	C13	1.321(6)
Tc2	Cl3	2.3292(10)		N7	N6	1.368(4)
Tc2	Cl4	2.3375(11)		N7	C8	1.308(5)
Tc2	O1	1.937(2)		N2	N1	1.375(4)
Tc2	O2	1.660(3)		N2	C3	1.318(5)
Tc2	N7	2.153(3)		N1	C5	1.300(5)
Tc2	N2	2.138(3)		N6	C10	1.303(5)
N4	C3	1.334(5)		N11	C15	1.300(6)
N4	C5	1.351(5)				

Table S5. Bond Angles for **2.**

Atom	Atom	Atom	Angle/°		Atom	Atom	Atom	Angle/°
Cl2	Tc1	Cl1	91.32(4)		N2	Tc2	Cl3	88.78(9)
O1	Tc1	Cl1	92.59(8)		N2	Tc2	Cl4	170.59(9)
O1	Tc1	Cl2	91.78(8)		N2	Tc2	N7	87.23(12)
O1	Tc1	N1	78.08(11)		Tc1	O1	Tc2	128.89(13)
O1	Tc1	N6	78.52(11)		C3	N4	C5	106.9(3)
O3	Tc1	Cl1	101.06(10)		C13	N12	N11	111.2(4)
O3	Tc1	Cl2	102.06(10)		C8	N9	C10	106.7(3)
O3	Tc1	O1	160.22(11)		C13	N14	C15	106.2(4)
O3	Tc1	N1	87.38(13)		N6	N7	Tc2	119.6(2)
O3	Tc1	N6	87.15(13)		C8	N7	Tc2	132.8(3)
N1	Tc1	Cl1	90.83(9)		C8	N7	N6	107.1(3)
N1	Tc1	Cl2	169.72(9)		N1	N2	Tc2	120.0(2)
N6	Tc1	Cl1	170.93(9)		C3	N2	Tc2	132.9(3)
N6	Tc1	Cl2	90.70(9)		C3	N2	N1	107.1(3)
N6	Tc1	N1	85.67(12)		N2	N1	Tc1	118.5(2)
Cl3	Tc2	Cl4	90.05(4)		C5	N1	Tc1	133.8(3)
O1	Tc2	Cl3	91.01(8)		C5	N1	N2	107.7(3)
O1	Tc2	Cl4	92.97(8)		N7	N6	Tc1	119.0(2)
O1	Tc2	N7	77.45(11)		C10	N6	Tc1	133.1(3)
O1	Tc2	N2	77.72(11)		C10	N6	N7	107.8(3)
O2	Tc2	Cl3	103.72(10)		C15	N11	N12	103.1(4)
O2	Tc2	Cl4	101.31(11)		N11	C15	N14	111.9(4)
O2	Tc2	O1	159.33(12)		N2	C3	N4	109.1(4)
O2	Tc2	N7	87.07(13)		N7	C8	N9	109.4(4)
O2	Tc2	N2	88.04(13)		N12	C13	N14	107.6(4)
N7	Tc2	Cl3	168.36(8)		N1	C5	N4	109.1(4)

N7	Tc2	Cl4	92.10(9)		N6	C10	N9	109.0(4)

Table S6. Hydrogen Bonds for **2.**

D	H	A	d(D-H)/Å	d(H-A)/Å	d(D-A)/Å	D-H-A/°
C13	H13	Cl3[1]	0.95	2.72	3.395(5)	128.5
C10	H10	Cl5[2]	0.95	2.76	3.384(4)	124.2
N14	H14	O1[1]	0.82(4)	2.01(4)	2.781(4)	157(4)
N9	H9	Cl5	0.77(4)	2.34(4)	3.100(4)	173(4)

[1]-X,1-Y,1-Z; [2]-1/2-X,-1/2+Y,1/2-Z

Table S7. Torsion Angles for **2.**

A	B	C	D	Angle/°		A	B	C	D	Angle/°
Tc1	N1	C5	N4	179.9(3)		N11	N12	C13	N14	0.1(6)
Tc1	N6	C10	N9	-175.5(3)		C15	N14	C13	N12	-0.5(6)
Tc2	N7	N6	Tc1	3.5(4)		C3	N4	C5	N1	-0.4(4)
Tc2	N7	N6	C10	-172.8(3)		C3	N2	N1	Tc1	179.9(2)
Tc2	N7	C8	N9	171.4(3)		C3	N2	N1	C5	-0.8(4)
Tc2	N2	N1	Tc1	-2.4(3)		C8	N9	C10	N6	-0.2(5)
Tc2	N2	N1	C5	176.9(2)		C8	N7	N6	Tc1	176.4(3)
Tc2	N2	C3	N4	-176.8(3)		C8	N7	N6	C10	0.0(4)
N12	N11	C15	N14	-0.7(5)		C13	N12	N11	C15	0.4(5)
N7	N6	C10	N9	0.1(4)		C13	N14	C15	N11	0.8(6)
N2	N1	C5	N4	0.7(4)		C5	N4	C3	N2	-0.1(5)
N1	N2	C3	N4	0.5(4)		C10	N9	C8	N7	0.2(5)
N6	N7	C8	N9	-0.1(4)						

Table S8. Bond Lengths for **3.**

Atom	Atom	Length/Å		Atom	Atom	Length/Å
Tc1	O1	1.69(4)		Tc2	O3	1.62(4)
Tc1	N1[1]	2.114(16)		Tc2	O2	1.95(7)
Tc1	N1[2]	2.114(16)		C3	C4	1.48(4)
Tc1	N1[3]	2.114(16)		C3	N2	1.31(3)
Tc1	N1	2.114(16)		C3	C2	1.40(4)
Tc1	O2	1.90(7)		N1	N2	1.37(3)
Tc2	Cl1[2]	2.397(5)		N1	C1	1.35(3)

Tc2	Cl1^{3}	2.397(5)		C2	C1	1.41(3)
Tc2	Cl1	2.397(5)		C1	C5	1.47(4)
Tc2	Cl1^{1}	2.397(5)				

^{1}1-Y,+X,+Z; ^{2}1-X,1-Y,+Z; ^{3}+Y,1-X,+Z

Table S9. Bond Angles for **3.**

Atom	Atom	Atom	Angle/°		Atom	Atom	Atom	Angle/°
O1	Tc1	N1^{1}	92.7(7)		O3	Tc2	Cl1^{3}	94.8(2)
O1	Tc1	N1^{2}	92.7(7)		O3	Tc2	Cl1	94.8(2)
O1	Tc1	N1	92.7(7)		O3	Tc2	Cl1^{1}	94.8(2)
O1	Tc1	N1^{3}	92.7(7)		O3	Tc2	Cl1^{2}	94.8(2)
O1	Tc1	O2	180.00(2)		O3	Tc2	O2	180.000(13)
N1^{2}	Tc1	N1^{3}	174.5(14)		O2	Tc2	Cl1^{2}	85.2(2)
N1^{1}	Tc1	N1^{3}	89.87(7)		O2	Tc2	Cl1	85.2(2)
N1^{2}	Tc1	N1	89.87(7)		O2	Tc2	Cl1^{3}	85.2(2)
N1^{1}	Tc1	N1	174.5(14)		O2	Tc2	Cl1^{1}	85.2(2)
N1^{1}	Tc1	N1^{2}	89.87(7)		N2	C3	C4	123(2)
N1	Tc1	N1^{3}	89.87(7)		N2	C3	C2	107(2)
O2	Tc1	N1^{3}	87.3(7)		C2	C3	C4	130(2)
O2	Tc1	N1^{1}	87.3(7)		N2	N1	Tc1	120.3(16)
O2	Tc1	N1^{2}	87.3(7)		C1	N1	Tc1	134.4(18)
O2	Tc1	N1	87.3(7)		C1	N1	N2	105.2(18)
Cl1^{2}	Tc2	Cl1^{3}	170.4(4)		C3	N2	N1	113(2)
Cl1^{1}	Tc2	Cl1^{2}	89.60(3)		C3	C2	C1	105(2)
Cl1^{1}	Tc2	Cl1	170.4(4)		N1	C1	C2	110(2)
Cl1	Tc2	Cl1^{3}	89.60(3)		N1	C1	C5	121(2)
Cl1^{1}	Tc2	Cl1^{3}	89.60(3)		C2	C1	C5	129(3)
Cl1^{2}	Tc2	Cl1	89.60(3)		Tc1	O2	Tc2	180.0

^{1}1-X,1-Y,+Z; ^{2}+Y,1-X,+Z; ^{3}1-Y,+X,+Z

Table S10. Hydrogen Bonds for **3.**

D	H	A	d(D-H)/Å	d(H-A)/Å	d(D-A)/Å	D-H-A/°
N2	H2	Cl1^{1}	0.88	2.38	3.24(2)	163.7
C5	H5A	N1^{1}	0.98	2.67	3.38(4)	130.4

^{1}1-Y,+X,+Z

Table S11. Torsion Angles for **3.**

A	B	C	D	Angle/°		A	B	C	D	Angle/°
Tc1	N1	N2	C3	-176.2(17)		C4	C3	C2	C1	175(3)
Tc1	N1	C1	C2	174.8(17)		N2	C3	C2	C1	-1(3)
Tc1	N1	C1	C5	-4(4)		N2	N1	C1	C2	-1(3)
C3	C2	C1	N1	2(3)		N2	N1	C1	C5	180(2)
C3	C2	C1	C5	-180(3)		C2	C3	N2	N1	0(3)
C4	C3	N2	N1	-176(3)		C1	N1	N2	C3	1(3)

Table S12. Bond Lengths for **4.**

Atom	Atom	Length/Å		Atom	Atom	Length/Å
Tc1	Cl1	2.371(3)		N9	C8	1.314(14)
Tc1	Cl2	2.450(3)		N1	C2	1.333(13)
Tc1	O1	1.861(8)		N1	C6	1.367(14)
Tc1	O2	1.650(8)		N7	C12	1.351(13)
Tc1	N1	2.132(9)		N7	C8	1.352(13)
Tc1	N7	2.144(9)		C5	C6	1.379(16)
O1	C13	1.429(14)		C5	C4	1.399(16)
N3	C2	1.348(15)		C11	C10	1.377(16)
N3	C4	1.357(15)		C11	C12	1.388(16)
N9	C10	1.350(14)				

Table S13. Bond Angles for **4.**

Atom	Atom	Atom	Angle/°		Atom	Atom	Atom	Angle/°
Cl1	Tc1	Cl2	174.86(10)		C2	N3	C4	116.3(10)
O1	Tc1	Cl1	90.7(3)		C8	N9	C10	115.1(10)
O1	Tc1	Cl2	84.1(3)		C2	N1	Tc1	120.3(8)
O1	Tc1	N1	90.6(3)		C2	N1	C6	118.3(10)
O1	Tc1	N7	87.6(3)		C6	N1	Tc1	121.4(8)
O2	Tc1	Cl1	97.2(3)		C12	N7	Tc1	121.6(7)
O2	Tc1	Cl2	87.9(3)		C12	N7	C8	116.9(10)
O2	Tc1	O1	171.0(4)		C8	N7	Tc1	121.4(7)
O2	Tc1	N1	93.7(4)		C6	C5	C4	118.7(11)
O2	Tc1	N7	88.1(4)		N1	C2	N3	125.6(10)
N1	Tc1	Cl1	89.6(3)		C10	C11	C12	117.3(10)
N1	Tc1	Cl2	90.5(3)		N9	C10	C11	123.4(11)
N1	Tc1	N7	178.2(3)		N7	C12	C11	120.3(10)
N7	Tc1	Cl1	90.2(3)		N1	C6	C5	119.8(10)

N7	Tc1	Cl2	89.6(3)		N9	C8	N7	127.0(11)
C13	O1	Tc1	148.1(7)		N3	C4	C5	121.3(11)

Table S14. Hydrogen Bonds for **4.**

D	H	A	d(D-H)/Å	d(H-A)/Å	d(D-A)/Å	D-H-A/°
C2	H2	O2[1]	0.95	2.42	3.222(13)	141.7
C8	H8	O1[2]	0.95	2.51	3.323(14)	143.9

[1]1+X,+Y,+Z; [2]-1+X,+Y,+Z

Table S15. Torsion Angles for **4.**

A	B	C	D	Angle/°		A	B	C	D	Angle/°
Tc1	N1	C2	N3	177.0(9)		C10	N9	C8	N7	-0.2(18)
Tc1	N1	C6	C5	-176.6(8)		C10	C11	C12	N7	-2.9(18)
Tc1	N7	C12	C11	-177.7(8)		C12	N7	C8	N9	-0.5(18)
Tc1	N7	C8	N9	179.3(10)		C12	C11	C10	N9	2.3(18)
Cl1	Tc1	O1	C13	-158.7(13)		C6	N1	C2	N3	0.1(18)
Cl2	Tc1	O1	C13	21.3(13)		C6	C5	C4	N3	-1.2(18)
N1	Tc1	O1	C13	111.7(14)		C8	N9	C10	C11	-0.8(18)
N7	Tc1	O1	C13	-68.5(14)		C8	N7	C12	C11	2.0(16)
C2	N3	C4	C5	1.5(17)		C4	N3	C2	N1	-0.9(18)
C2	N1	C6	C5	0.2(16)		C4	C5	C6	N1	0.3(17)

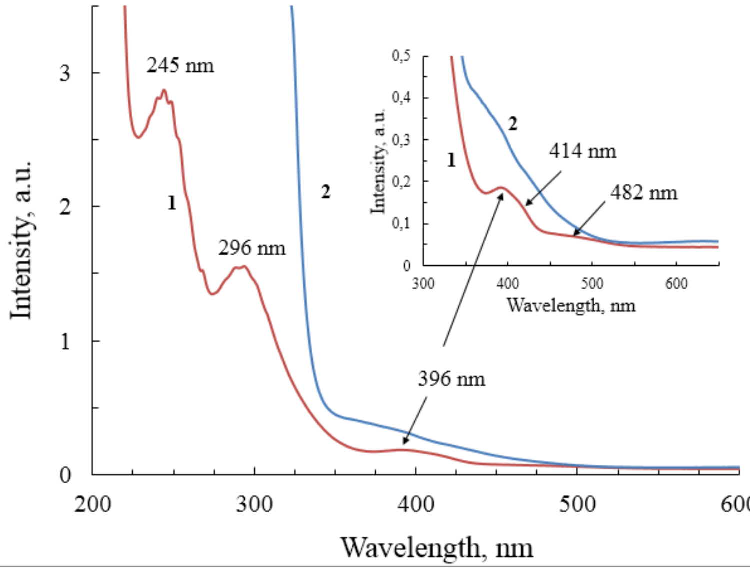

Figure S1. Electron absorption spectroscopy of Tc(V) in methanol solution - 1, in acetone solution - 2.

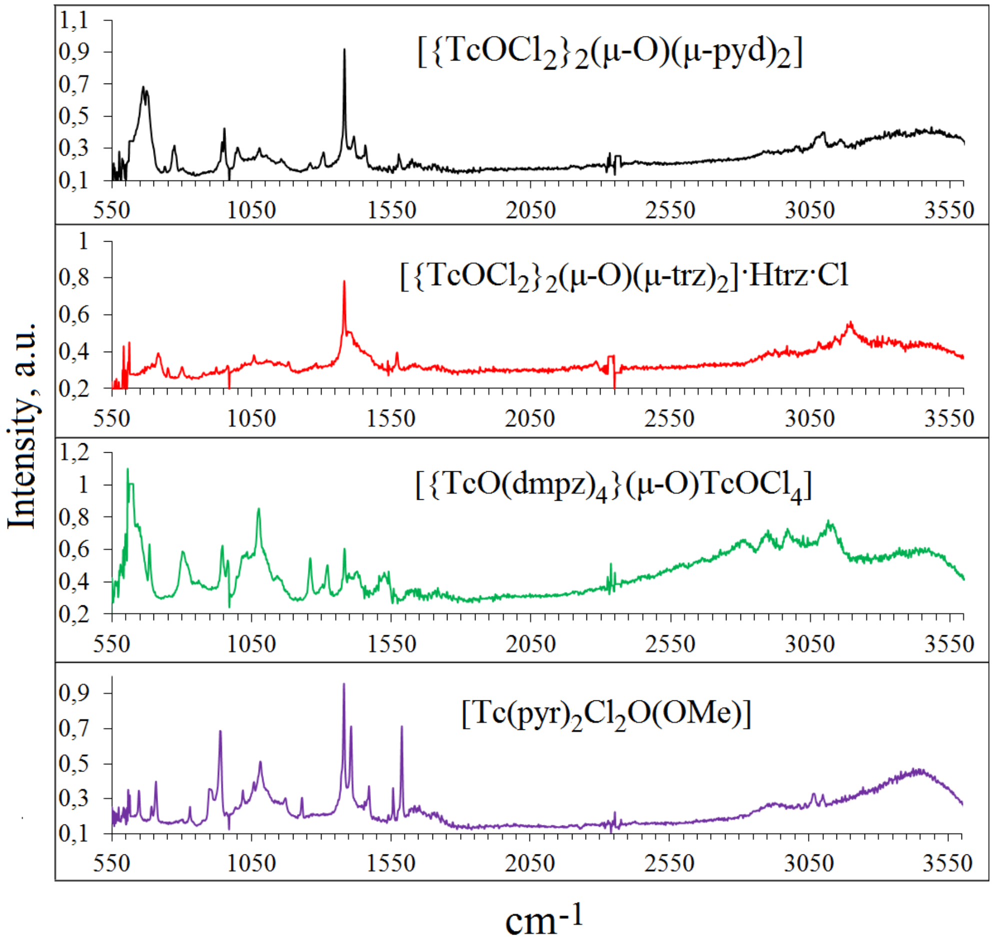

Figure S2. IR spectroscopy of complex compounds.

Table S16. List of IR peaks.

Complex **1**		Complex **2**		Complex **3**		Complex **4**	
Peak, cm^{-1}	Intensity, a.u.	Peak, cm^{-1}	Intensity, a.u.	Peak, cm^{-1}	Intensity, a.u.	Peak, cm^{-1}	Intensity, a.u.
576	0.27	592	0.43	606	1.09	592	0.25
622	0.34	612	0.45	626	1.00	608	0.35
676	0.65	722	0.37	650	0.74	646	0.35
680	0.62	754	0.28	684	0.63	694	0.23
736	0.13	804	0.39	810	0.56	708	0.40
776	0.28	926	0.32	858	0.39	814	0.16
814	0.15	968	0.31	944	0.61	830	0.25
936	0.21	1062	0.36	966	0.52	910	0.30
952	0.42	1186	0.32	1004	0.43	940	0.68
1006	0.30	1286	0.32	1014	0.54	1006	0.24
1080	0.29	1372	0.78	1056	0.58	1020	0.33
1170	0.20	1410	0.49	1086	0.75	1058	0.39
1266	0.16	1486	0.35	1100	0.58	1087	0.47
1310	0.25	1520	0.31	1158	0.40	1102	0.35

1382	0.91	1538	0.35	1260	0.54	1170	0.30
1412	0.36	1572	0.39	1320	0.49	1230	0.29
1460	0.29	1644	0.31	1482	0.60	1382	0.97
1580	0.24	1720	0.31	1436	0.44	1408	0.71
1628	0.21	2290	0.33	1456	0.37	1472	0.37
1698	0.29	2340	0.37	1528	0.43	1554	0.21
2352	0.25	2928	0.40	1558	0.35	1558	0.36
2362	0.25	2986	0.43	1644	0.32	1590	0.71
2878	0.27	3082	0.47	1716	0.36	1640	0.23
3092	0.39	3140	0.48	2324	0.42	1720	0.21
-	-	3196	0.56	2336	0.51	2328	0.17
-	-	3310	0.48	2350	0.45	2354	0.22
-	-	-	-	2826	0.64	2380	0.16
-	-	-	-	2908	0.69	2938	0.27
-	-	-	-	2968	0.72	3062	0.31
-	-	-	-	3114	0.78	3102	0.31

Table S17. Interpretation of MALDI-ToF peaks.

<table>
<tr><th rowspan="2">m/z</th><th rowspan="2">Possible structure</th><th colspan="2">Isotope distribution</th></tr>
<tr><th>experiment</th><th>theoretical</th></tr>
<tr><td>Calc.
246.95</td><td rowspan="2">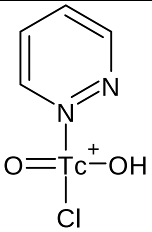</td><td rowspan="2">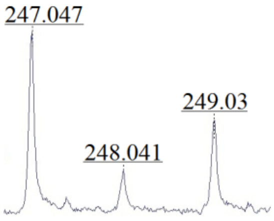</td><td rowspan="2">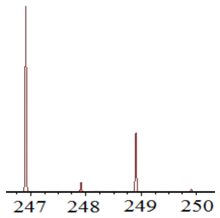</td></tr>
<tr><td>Found
247.047</td></tr>
<tr><td>Calc.
281.98</td><td rowspan="2">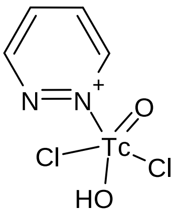</td><td rowspan="2">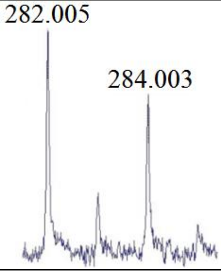</td><td rowspan="2">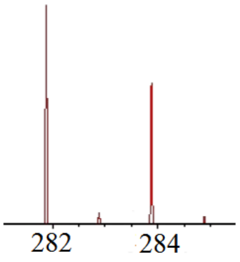</td></tr>
<tr><td>Found
282.005</td></tr>
</table>

Calc.<br>344.97<br>Found<br>344.994	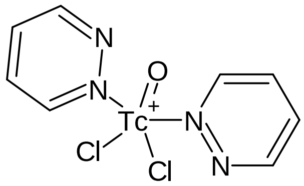	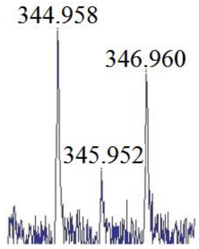	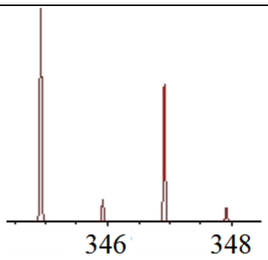
Calc.<br>361.99<br>Found<br>362.043	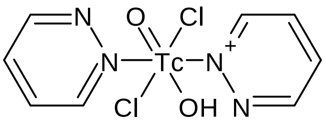	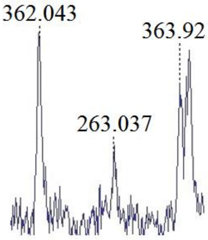	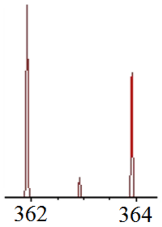
Calc.<br>462.75<br>Found<br>462.82	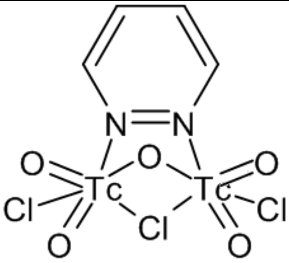	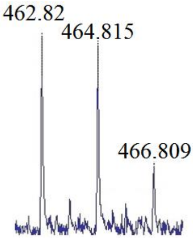	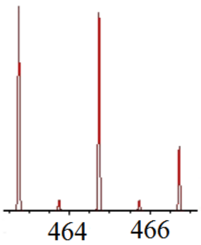
Calc.<br>499.777<br>Found<br>499.784	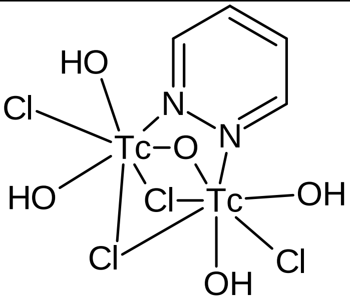	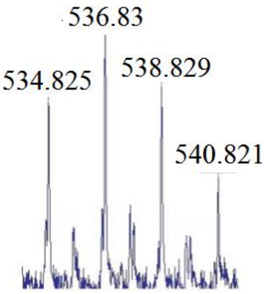	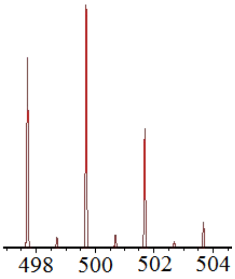
Calc.<br>536.79<br>Found<br>536.83	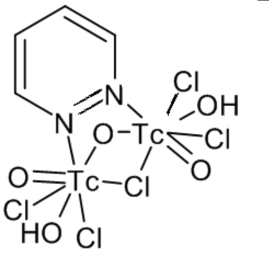	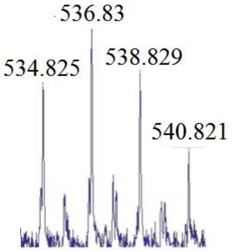	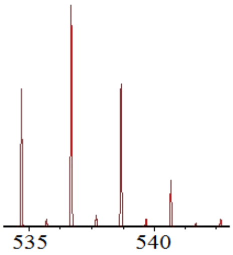

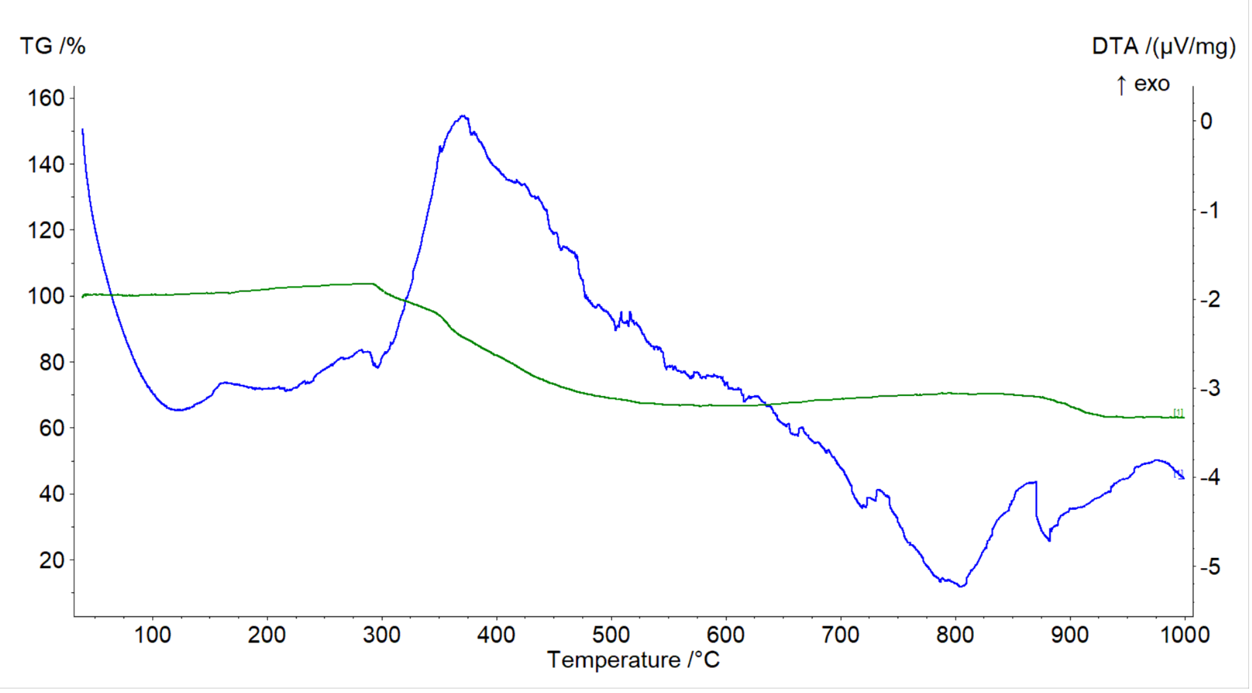

Figure S3. TG-DTA of compound **1**.

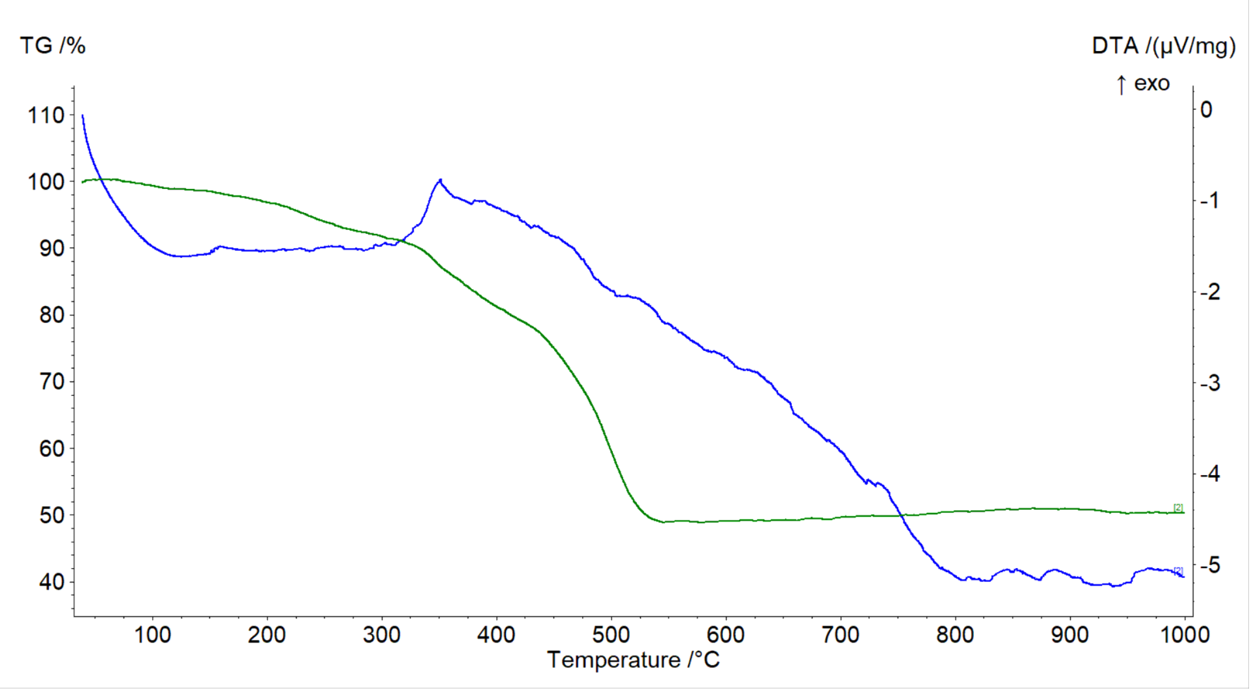

Figure S4. TG-DTA of compound **2**.
